# Design, Preparation, and Characterization of Dioscin Nanosuspensions and Evaluation of Their Protective Effect against Carbon Tetrachloride-Induced Acute Liver Injury in Mice

**DOI:** 10.1155/2019/3907915

**Published:** 2019-11-14

**Authors:** Hong-Ye JU, Kun-Xia Hu, Guo-Wang Zhao, Zhi-Shu Tang, Xiao Song

**Affiliations:** School of Pharmacy, Shaanxi University of Chinese Medicine, Xianyang 712046, China

## Abstract

The purpose of this study was to prepare a dioscin nanosuspension (Dio-NS) that has a better distance and high solubility for oral administration and to evaluate its hepatoprotective effects. Optimal primary manufacture parameters, including shear time, shear speed, emulation temperature, pressure, and cycles of homogenization, were determined by single-factor experiments. The concentrations of dioscin, SDS, and soybean lecithin were optimized using the central composite design-response surface method, and their effects on the mean particle size (MPS) and particle size distribution of Dio-NS were investigated. Characterization of the Dio-NS formulations included examinations of the surface morphology and physical status of dioscin in Dio-NS, the stability of Dio-NS at different temperatures, *in vitro* solubility, and liver protective effect *in vivo*. Under optimal conditions, Dio-NS had an MPS of 106.72 nm, polydispersity index of 0.221, and zeta potential of −34.27 mV. Furthermore, the proportion of dioscin in Dio-NS was approximately 21.26%. The observation of particles with a spherical shape and the disappearance of crystalline peaks indicated that the physical and chemical properties of Dio-NS were altered. Furthermore, we observed that the dissolution of Dio-NS was superior to that of a physical mixture and Dio-GZF. Moreover, Dio-NS was demonstrated to have a protective effect against CCl_4_-induced acute liver damage in mice that was equivalent to that of silymarin (a positive control drug) at the same dose. The good hepatoprotective effect of our Dio-NS preparation can provide a theoretical basis for investigating its absorption mechanisms in the body.

## 1. Introduction

**Figure 1:**
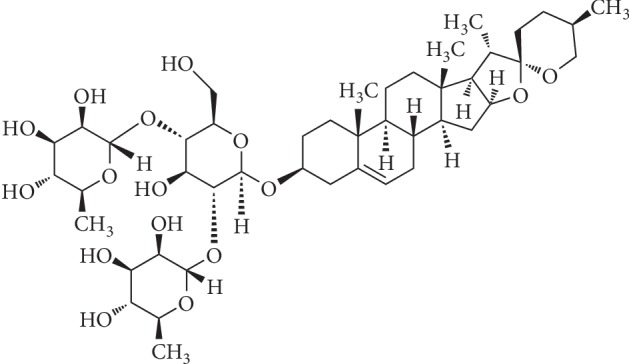
The chemical structure of dioscin.

Dioscin (Figure 1), also known as *Paris polyphylla* saponin III, is a steroidal saponin [1]that can be extracted from the Chinese yam (*Dioscorea paniculata*) and other plants in the Dioscoreaceae family [2]. Dioscin has several important pharmacological activities, including antitumor [3], anti-inflammatory [4], antiliver damnification [5], antihepatic fibrosis [6, 7], antihyperlipidemic, and antioxidative properties. Furthermore, the compound has therapeutic potential in metabolic diseases and can also be decomposed into diosgenin, which has been an important basic raw material for the production of steroid hormone drugs [8].Meanwhile, it has also been established that there is potential to develop the pharmaceutic value of dioscin. In addition, some reports have been found on dioscin toxicology. It has no adverse effect on the acute toxicological studies at a dose of 562.5 mg/kg/d in mice [9, 10]. However, on the subchronic toxicological assessment, it was verified as the NOAEL (no-observed-adverse-effect level) at a dose of 300 mg/kg/day in female rats and in male rats was marked as the LOAEL (lowest-observed-adverse-effect level) at the same dose [11]. So, rational drug use is the key to lower ADR.

In fact, dioscin has a notable disadvantage, in that when the valid route fails, the probability of a new established route occurring via secondary chain scission is large. Its poor solubility and slow dissolution rate limit its absorption, thus affecting its efficacy.

Poor solubilities and dissolution rates are key factors affecting the absorption of drugs in the body. Thus, several techniques have been devised in order to overcome these undesirable properties, including micronization [12], solubilization, and salt formation [13]. However, these techniques are only partially effective. The advent of nanotechnology, which can produce nanoscale particles with novel functional properties, offers a potential solution to the aforementioned drawbacks. Nanotechnology has, for example, been used to enhance the bioavailability of certain antibiotics [14] and has also been applied to produce different targeted drugs for the treatment of different diseases [15–17], and various nanosuspensions have been successfully applied [18, 19]. Nanoparticles can also be prepared using a variety of different processes, including reverse solvent, homogenization pressure, milling, and the production of self-nanoemulsifying self-nanosuspensions [20].

Diseases of the liver, including hepatitis, hepatapostema, hepatocirrhosis, and liver cancer, are common and often life-threatening. Carbon tetrachloride (CCl_4_) [21, 22], paracetamol [23, 24], lipopolysaccharide [25, 26], and certain other chemicals can also cause acute liver damage, and consequently, a large number of drugs are used for the treatment of hepatic disorders [27]. It was confirmed that dioscin has significant hepatoprotective effect against CCl4-induced liver injury in mice, and the cellular mechanisms of this effect are likely to be associated with inhibition of lipid peroxidation, inflammatory cytokines, necrosis, and apoptosis. Thus, in the present study, we formulated a dioscin nanosuspension (Dio-NS) using reverse solvent precipitation combined with high-pressure homogenization and compared its efficacy with that of the commercial drug silymarin in protecting mice from acute hepatic damage induced by CCl_4_. We assumed that the Dio-NS formulation would enhance the solubility of dioscin and thus increase the efficacy of this compound in protecting against liver injury.

## 2. Methods

### 2.1. Materials

Dioscin was purchased from Nanjing Spring and Autumn Biological Engineering (Nanjing, China) with the purity of >99%. Soybean lecithin, mannitol, poloxamer-188, Tween-80, PVP K30, sodium cholate, sodium alkyl sulfate twelve (SDS), anhydrous ethanol, lactose, glucose, sucrose, d-sorbitol, silymarin, 4% paraformaldehyde solution, methanol, and acetonitrile were all of chromatographic grade, and the other reagents were all of analytical grade. AST and ALT kits were purchased from Nanjing Jiancheng Institute of Biotechnology (Nanjing, China). TNF-*α*, IL-1*β*, IL-6, MDA, SOD, and GSH-Px kits were purchased from Shanghai Elisa Biotech Co., Ltd.

### 2.2. Preparation of Dio-NS

**Figure 2:**
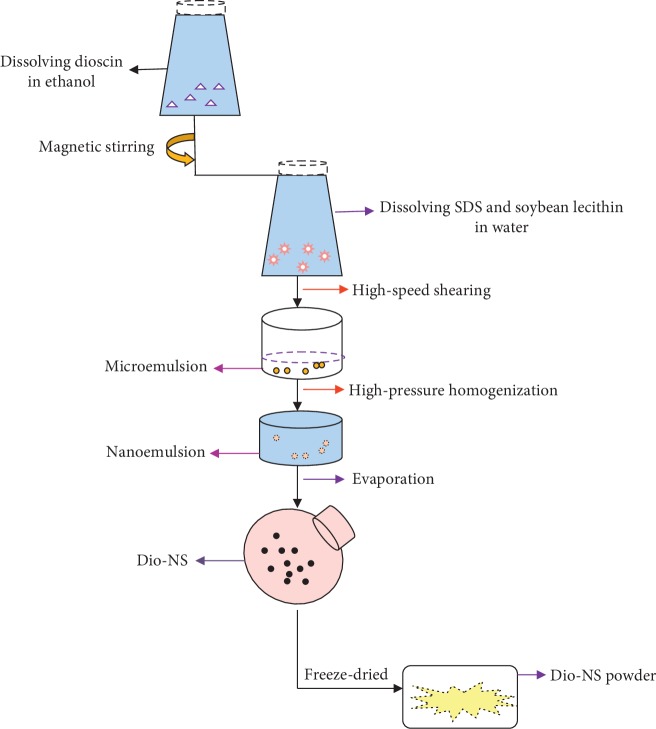
A flow diagram showing the steps involved in the preparation of Dio-NS.

Dioscin was dissolved in ethanol as a solvent and dispersed for 15 min in a KQ-3-DE ultrasonic bath (Kunshan, China). In the next emulsification process, ethanol was used as the organic phase of the emulsion. The solution was rapidly added dropwise to double-distilled water-saturated ethanol together with sodium alkyl sulfate twelve and soybean lecithin as excipients that can be used to improve the stability of emulsions, commixing using magnetic stirrer (HJ-6B, Changzhou, China). A microemulsion was obtained by high-speed shearing (FJ-200, ShenZhen, China). The microemulsion preparation was then homogenized to produce a nanoemulsion via high-pressure homogenization (AH-BASICI, ATS Engineering Inc., Canada). An EYELA N-1300 rotary evaporator was used to remove the ethanol (LangYi, Shanghai, China) at an evaporation temperature of 40°C, thereby yielding the Dio-NS preparation. In this study, the optimal process conditions were determined on the basis of single-factor experimental results. All the preparation steps are shown in Figure 2.

### 2.3. Preparation of a Physical Mixture

The desired dosage of Dio-NS was obtained by dissolving in water and freeze-drying for 12 h to yield an NS-A physical mixture.

### 2.4. Screening of Excipients

Eleven groups were examined to select the best accessories that have a minimum size and powder dispersion index.

### 2.5. Single-Factor Experiment

In the single-factor experiment, particle size and multiple dispersion index were taken as indicators to determine optimal preparation parameters, including shear time, shear speed, emulation temperature, pressure, and cycles of homogenization.

### 2.6. Optimization of the Response Surface of the Star Design

On the basis of the results of the single-factor experiment and the feasibility of preparing nanosuspensions at the highest or lowest level, we selected the level of each factor and the star response surface design software and determined the best experimental results.

### 2.7. Lyophilization

The Dio-NS preparation was lyophilized using a VaCo-5 freeze-drying apparatus (Zirbus, Germany) to obtain dry powder with good physicochemical stability. In this study, a single-factor method was used to select optimal conditions, and the best freeze-drying protectant was selected. The results are shown in Table 1. The content of freeze-drying protectant ranged from 2% to 10%. The levels are shown in Table 2.

### 2.8. Particle Size and Zeta Potential

The mean particle size (MPS), particle size distribution (PSD), and zeta potential of the formulation were determined using a Malvern particle size meter. Samples were prepared by dissolution of Dio-NS in deionized water, and each property was measured 3 times.

### 2.9. Morphological Observations (SEM)

The morphology of particles was determined by scanning electron microscopy (SEM) (MERLIN Compact, German). Samples were affixed to aluminum stubs using a double-sided carbon tape and sputter-coated with gold under an argon atmosphere.

### 2.10. Transmission Electron Microscopy (TEM)

After 4 types of samples had been vortexed for 2 min, they were dripped onto a copper sheet of a transmission electron microscope (JEOL, Japan), and the excess moisture was dried using a filter paper. Images of the samples were observed on the perspective electron microscope.

### 2.11. X-Ray Powder Diffraction

The crystalline state of substances was typically determined by X-ray powder diffraction. Diffraction patterns of the Dio-NS and other auxiliary material were determined using a Bruker D8 advance X-ray diffractometer (Bruker, Germany). A Cu line was used as the source of radiation, and standard runs were performed at a voltage of 40 kV, step length of 0.01°, current of 40 mA, and a scanning rate of 0.1 s/step over a 2 theta range of 5–90°.

### 2.12. Thermal Analysis

Individual samples were placed in different aluminum differential scanning calorimeter (DSC) pans (214 Polyma, Netzsch, Germany), which were heated to 200°C and 150°C for approximately 15 min. Thereafter, the samples were quench-cooled in liquid nitrogen externally to the DSC instrument. DSC was implemented by returning the samples to a prerefrigerating room (−50°C), and thermograms were recorded.

### 2.13. In Vitro Dissolution of Dio-NS

The Dio-NS powder and physical mixture were loaded into dialysis membranes, which were placed in constant-speed oscillators at constant temperature and oscillated for different times. Samples of Dio-NS obtained at different time points were analyzed by HPLC (Waters e2695, USA). The cumulative release rate was determined using the following equation:

(1) $$\begin{array}{c} Q\%\left(\text{cumulative release rate}\right)=\frac{\left(Qt_{1}+Qt_{2}+\cdots +Qt_{n}\right)}{Q}\times 100\%, \end{array}$$

where *Qt*_1_,*Qt*_2_,…,*Qt*_n_ is the content of dioscin dissolved in Dio-NS between time *t*_0_ and *t*_1,2,…,*n*_.

### 2.14. Stability Study

The stability of the dioscin nanoscale suspension at temperatures of 4°C and 25°C was investigated over a period of 1 month, and the results are shown in Table 3.

### 2.15. The Effects of Dio-NS on Liver Preservation

#### 2.15.1. Animals and Treatment

Kunming male mice (18–22 g) were purchased from Chengdu Dashuo Biotechnology Co., Ltd. (Chendu, China.). The mice were acclimated for 1 week in a controlled environment at 25 ± 2°C under a 12 h dark/light photoperiod. They were subsequently randomly divided into the following 6 groups each containing 8 animals: group 1 (normal control) and group 2 (model control) were orally administered water for 7 days; group 3 (positive control) received silymarin (50 mg/kg) for 7 days; and groups 4 to 6 (low-, medium-, and high-dose groups, respectively) were administered Dio-NS (25, 50, and 100 mg/kg, respectively) intragastrically. Two hours after the final administration, the animals in group 1 were injected intraperitoneally with pure olive oil, whereas mice in the other groups were treated with 0.2% (v/v) CCl_4_ (10 mL/kg body weight, i.p.; dissolved in olive oil) [28]. Thereafter, the mice were maintained with free access to water and were sacrificed 24 h later, at which time blood was collected for the preparation of serum. After killing the animals, the livers were immediately isolated and weighed to determine the liver index (liver index = liver weight/body weight × 100%). A portion of each liver was fixed in 4% paraformaldehyde solution for pathological study, and the remainder along with the acquired serum samples were stored at −80°C for subsequent studies. The entire process was performed on ice.

#### 2.15.2. Serum Biochemistry Assays

The activities of ALT, AST, TNF-*α*, IL-1*β*, and IL-6 in the serum were measured using commercial kits following the manufacturer's instructions.

#### 2.15.3. Liver Lipid Peroxidation Assay

After saline was added to the appropriate liver tissue, the sample was placed into an automated tissue homogenizer (SCIENTZ-48, Ningbo, China) to prepare a 10% hepatic homogenate. Samples were processed using commercial kits according the manufacturer's instructions, and the levels of MDA, GSH-Px, and SOD in the liver homogenate were evaluated using a microplate reader.

#### 2.15.4. Histopathological Examination

Fixed liver tissues were embedded in paraffin, from which 5-*µ*m sections were cut and placed on slides. The samples were stained with hematoxylin and eosin (H&E) and then observed at ×400 magnification.

#### 2.15.5. Statistical Analysis

SPSS version 19.0 statistical software was used to analyze all the data using a one-way ANOVA. Data are represented as mean ± S.E.M. Differences between groups were considered significant at *p* < 0.01 and *p* < 0.05 levels.

## 3. Results

### 3.1. Evaluation of the Dio-NS Preparation

The results of stabilizer screening showed that different stabilizers had a considerable influence on the average particle size, polydispersity index, and system storage stability of drug suspensions [29](Table 4). On the premise of maintaining the same total amount, the combined use of different types of stabilizers effectively reduced the 3 qualitative indexes of drug suspension. In this study, we found that when the combined application of soybean lecithin and SDS was used as a stabilizer for the suspension system, the average size of the drug particles was small, the distribution was narrow, and the physical stability of the system was good. Therefore, we used soybean lecithin and SDS as stabilizers for Dio-NS. An SDS : lecithin ratio of 1 : 1 proved to be the optimal combination.

**Figure 3:**
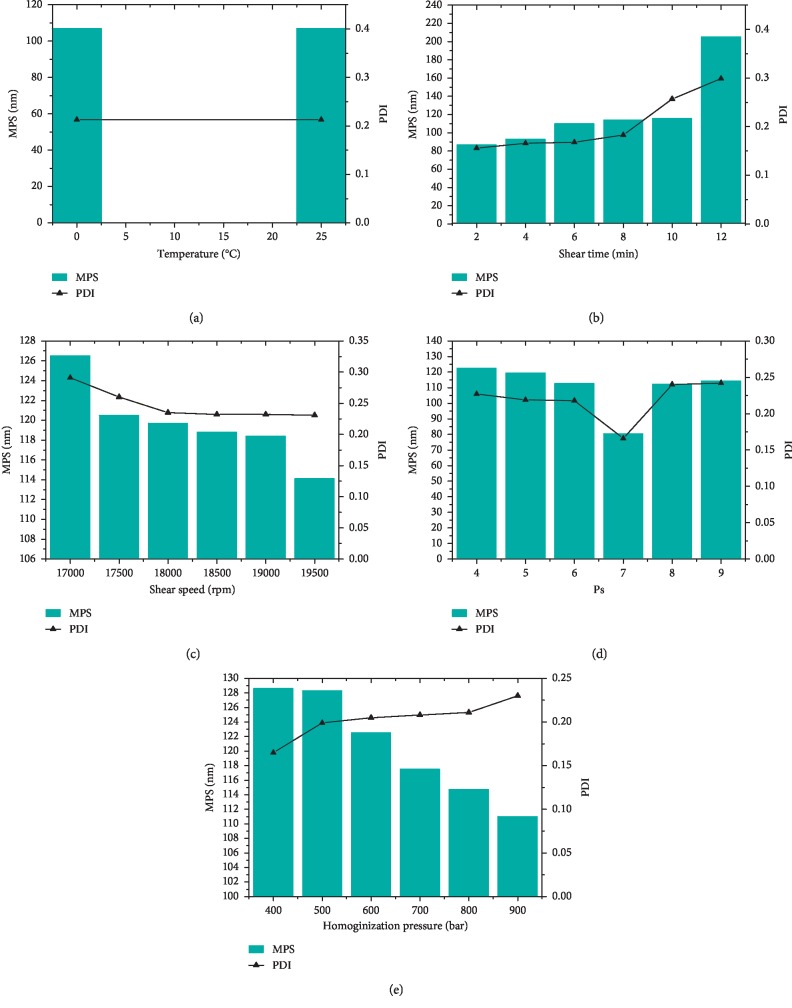
The influences of temperature (a), number of homogenization cycles (b), shear speed (c), shear time (d), and homogenization pressure (e) on the particle size and polydispersity index of Dio-NS preparations.

As shown in Figure 3, it is clear that temperature has little effect on emulsification. We also established that the minimum particle size and polydispersity index are obtained under the following conditions: a shear speed of 19,500 rpm, a shear time of 2 min, a homogeneous pressure of 800 bar, and 7 cycles of homogenization. We thus obtained the optimal operation parameters (Table 5).  Table 6 shows the results of 20-run experiments designed on the basis of 3 factors and 4 levels using Design-Expert 8.0.6 software. The results of a regression analysis are shown in equations (2) and (3):

(2) $$\begin{array}{c} Y=+0.66-0.095A-0.023B-0.065C+0.10AB-0.033AC+2.375E-003BC-0.13A^{2}+0.030B^{2}+0.047C^{2},R=0.9411,P<0.0001. \end{array}$$

OD = (*d*1,*d*2,…,*dk*)1^*K*^, where *K* is the number of indices.

(3) $$\begin{array}{c} d_{\min}=\left(Y_{\max}-Y_{i}\right)\left(Y_{\max}-Y_{\min}\right),\\ d_{\text{max}}=\left(Y_{i}-Y_{\min}\right)\left(Y_{\max}-Y_{\min}\right). \end{array}$$

**Figure 4:**
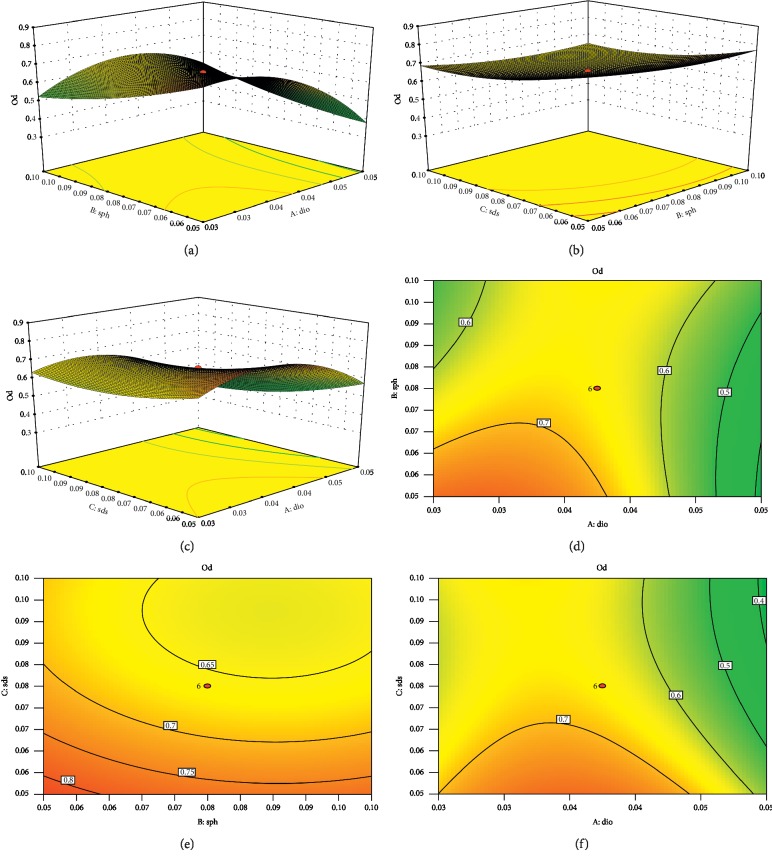
The response surface model of the reciprocal action among the concentrations of dioscin% (A), soybean lecithin (B), and SDS (C) on OD.

Figure 4 shows the results of the interaction between 2 groups. For Dio-NS preparation with a content of 26.67% (w/w) dioscin, 20% (w/w) SDS, and 53.33% (w/w) soybean lecithin, we obtained a maximum OD of 0.868, which was close to that predicted. As shown in Table 1, the diameter of Dio-NS dry powder particles, polydispersity index, and zeta potential were less than those obtained using other freeze-drying protection agents. Furthermore, the powder showed no collapse or shrinkage, the color and luster were more uniform, and the preparation had good dispersion, and therefore, soybean lecithin and SDS were selected as the protectants for the dioscin freeze-dried powder.

It can be seen from Table 2 that the particle size obtained using mannitol in the aqueous phase was 7%, and values for the polydispersity index and the zeta potential were the smallest recorded.

**Figure 5:**
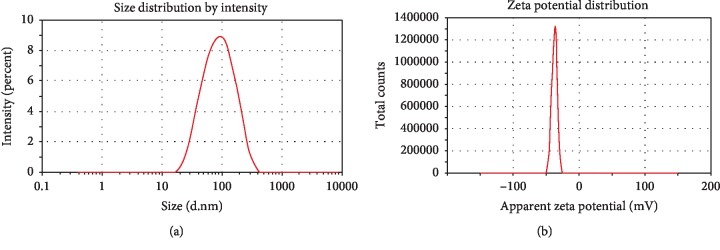
Particle size and zeta potential distribution of Dio-NS formulations.

The size, zeta potential, and polydispersity index of the Dio-NS formulations are shown in Table 7. A particle size diagram and zeta potential are shown in Figures 5(a) and 5(b), respectively. A sharp peak of Dio-NS, which represents its size, was identified at approximately 106.72 ± 8.90. Furthermore, the zeta potential of Dio-NS was −34.27 ± 2.91 mV, indicating the reasonable stability of the preparation.

### 3.2. Characterization of Dio-NS

#### 3.2.1. Particle Size and Zeta Potential

The particle size and zeta potential of the optimum Dio-NS are given in Table 7.

#### 3.2.2. Surface Morphology and Particle Size of Dio-NS

**Figure 6:**
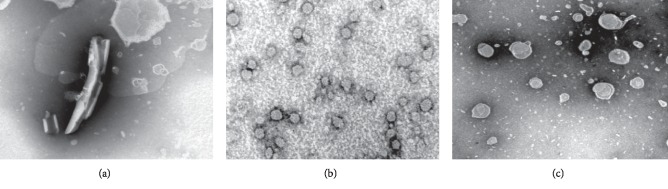
Transmission electron micrographs of samples dispersed in water. (a) Dioscin. (b) Dio-NS. (c) Dio-NS powder.

Transmission and scanning electron microscopy were applied to analyze the morphology and structure of the Dio-NS formulations. Dioscin has a tubular crystal structure (Figure 6(a)).  Figure 6 shows that Dio-NS has a near-spheroid particle structure with a particle size of approximately 106 nm.  Figure 6(b) shows that the redispersibility of Dio-NS powder in purified water was also nearly globoid and that the particle size is somewhat larger at approximately 173 nm.

**Figure 7:**
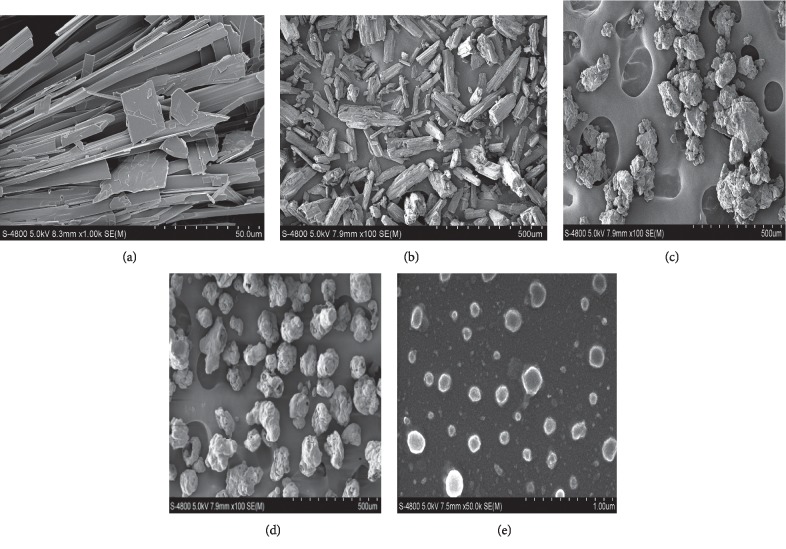
Scanning electron micrographs of (a) dioscin raw material, (b) with mannitol, (c) with soybean lecithin, (d) with twelve alkyl sulfates, and (e) Dio-NS lyophilized powder.

The scanning electron microscopy images shown in Figure 7 show that dioscin raw material (A) is acicular with a larger particle size, whereas that with mannitol B is an irregular prism, that with soybean lecithin (C) shows irregular lumps, and that with twelve alkyl sodium sulfate (D) is irregular spherical and loose with pores. Dio-NS nanoparticles (E) show the same shape as seen under transmission electron microscopy, and the morphology changes. As described above, these features indicate that the freeze-drying process has significant effect on particle size [30].

#### 3.2.3. Physical Status of Dioscin in the Dio-NS

**Figure 8:**
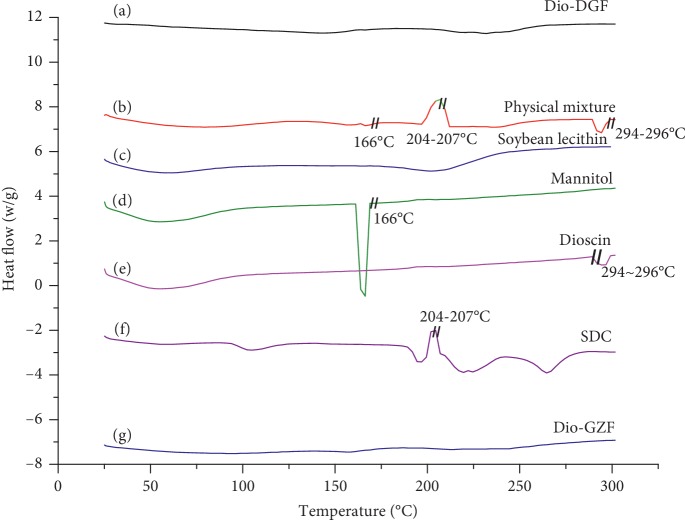
DSC patterns of freeze-dried Dio-NS (a), physical mixture (b), soybean lecithin (c), mannitol (d), raw dioscin (e), SDS (f), and dry Dio-NS (g).

DSC thermograms of pure dioscin, SDS, soybean lecithin, mannitol, physical mixture, and freeze-dried and dry powder of Dio-NS are shown in Figure 8. The DSC thermograms of raw dioscin, mannitol, and SDS show a sharp endothermic peak at approximately 294–296°C, 166°C, and 204–207°C, respectively. A peak similar to that of pure dioscin was also observed in the thermogram of the physical mixture, indicating a weak or negligible influence. However, this peak was not present in the freeze-dried and dry Dio-NS preparations, which indicates that dioscin occurs in an amorphous form in Dio-NS.

**Figure 9:**
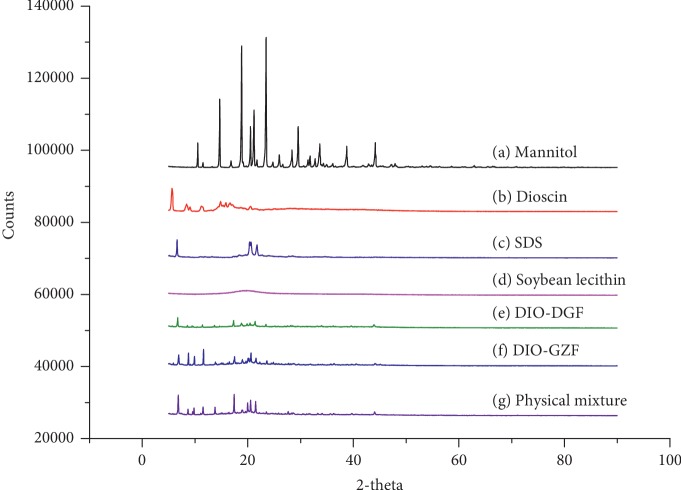
X-ray diffraction patterns of mannitol (a), raw dioscin (b), SDS (c), soybean lecithin (d), freeze-dried Dio-NS (e), dry Dio-NS (f), and physical mixture (g).

The diffraction intensity and distribution of each crystal will have a special rule [31]. By using X-ray diffraction, we can analyze whether the crystalline form has changed in the preparation process. The diffraction peak of dioscin raw powder shown in Figure 9 indicates that it has a strong crystalline structure, whereas the diffraction peak of the excipient soybean lecithin indicates that its structure is essentially amorphous. The peaks of dioscin in the physical mixture and dry powder are both in the crystal form of the carrier. The structure did not change, and the crystalline peak of the drug in the Dio-NS freeze-dried powder essentially disappeared, indicating that the drug occurs in an amorphous state and the freeze-drying process has an effect on the crystalline structure of the drug.

#### 3.2.4. In Vitro Dissolution and Stability

**Figure 10:**
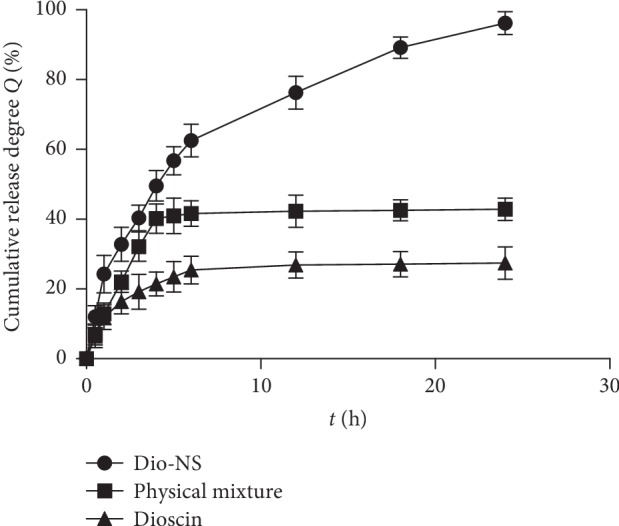
*In vitro* dissolution of dioscin nanosuspensions.

As shown in Figure 10, the dissolution rate of dioscin can be significantly improved after the preparation of nanosuspensions. In PBS (pH 7.4), the dissolution rate of Dio-NS during the initial 2 h was close to 30%, whereas the release rate at 12 h was close to 90%. The dissolution rate of the physical mixture group was slightly faster than that of the raw material group, which can be attributed to the solubilization effect of the twelve alkyl sulfate and soybean lecithin used in the formulation. However, the dissolution rate was considerably less than that of Dio-NS. It is suggested that the dissolution rate of nanosuspensions is not only caused by the solubilization of excipients but is also mainly due to the reduction of particle size after the formation of nanoscale suspensions.

**Figure 11:**
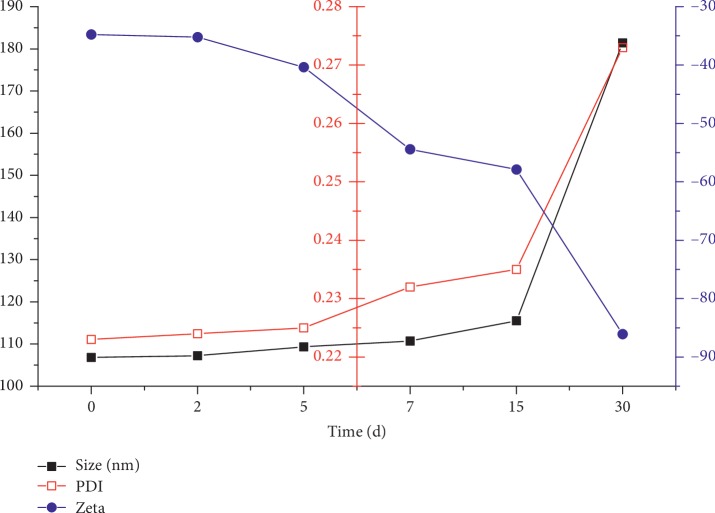
Particle size, polydispersity index, and zeta potential of dioscin nanoscale suspension NS-B at 25°C.

**Figure 12:**
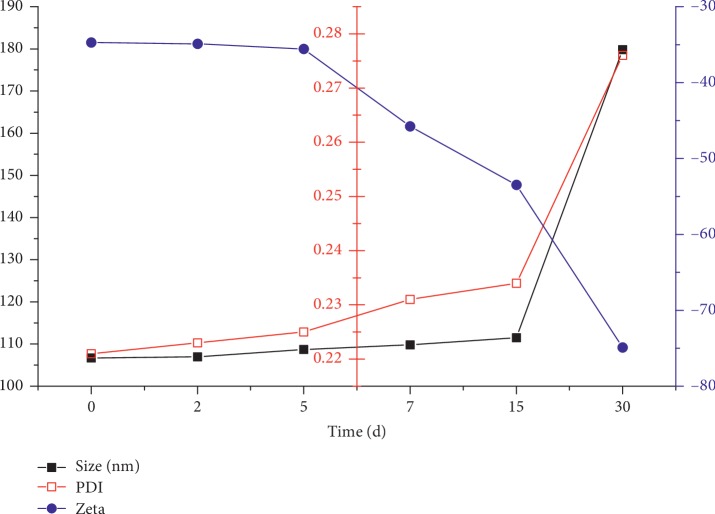
Particle size, polydispersity index, and zeta potential of dioscin nanoscale suspension NS-B at 4°C.

The results showed that although the non freeze-dried Dio-NS had poor stability at room temperature (Figures 11 and 12), the freeze-dried powder remained stable for 2 months (Table 3).

### 3.3. The Effects of Dio-NS on Liver Preservation

#### 3.3.1. Serum Biochemistry and Hepatic Lipid Peroxidation Assays

**Figure 13:**
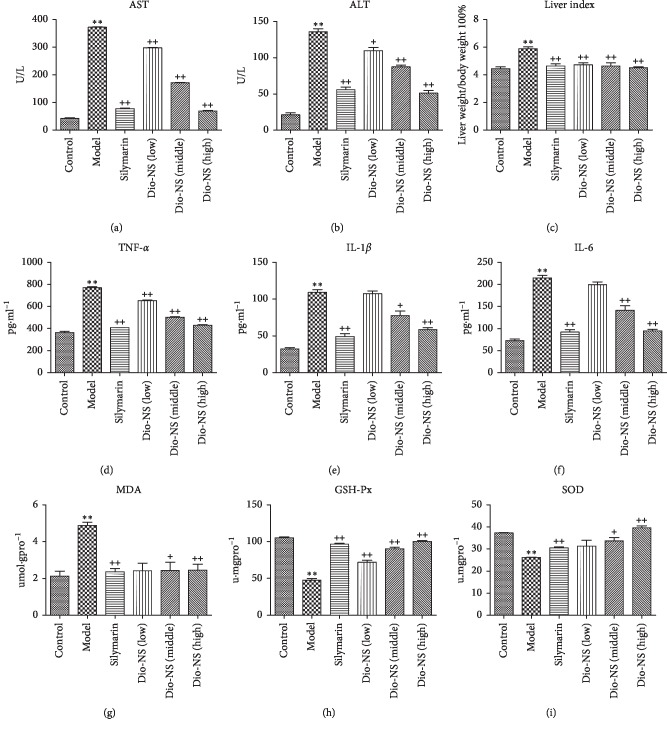
Effect of Dio-NS on CCl4-induced hepatotoxicity. (a) The activities of ALTin the serum. (b) The activities of ASTin the serum. (c) The liver index. (d) The activities of TNF-a in the serum. (e) The activities of IL-1β in the serum. (f ) The activities of IL-6 in the serum. (g) The levels of MDA in the liver. (h) The levels of GSH-px in the liver. (i) The levels of SOD in the liver. ^∗∗^*P* < 0.01 vs control, +*P* < 0.05, and ++*P* < 0.01 vs. model.

In order to assess the influence of Dio-NS on CCl_4_-induced liver damage, the activities of ALT, AST, TNF-*α*, IL-1*β*, and IL-6 in the serum were monitored (Figure 13). The results indicate that our model is successful, given that the model groups show significant difference compared with the control (*p* < 0.01). Compared with the model groups, there were significant differences in groups with different Dio-NS and silymarin doses (*p* < 0.01 and *p* < 0.05).

The results of our analysis of liver lipid peroxidation showed that the activity of GSH-Px and SOD in different dose groups of Dio-NS were markedly increased, compared with those of the model control (*p* < 0.01 and *p* < 0.05) (Figure 13). In contrast, the level of MDA was significantly reduced (*p* < 0.01 and *p* < 0.05). In the different drug groups, the liver index was reduced to some extent. Consequently, there was an equivalent or even better effect from the high-dose group (100 mg/kg) of Dio-NS compared with that of silymarin (the positive control drug).

**Figure 14:**
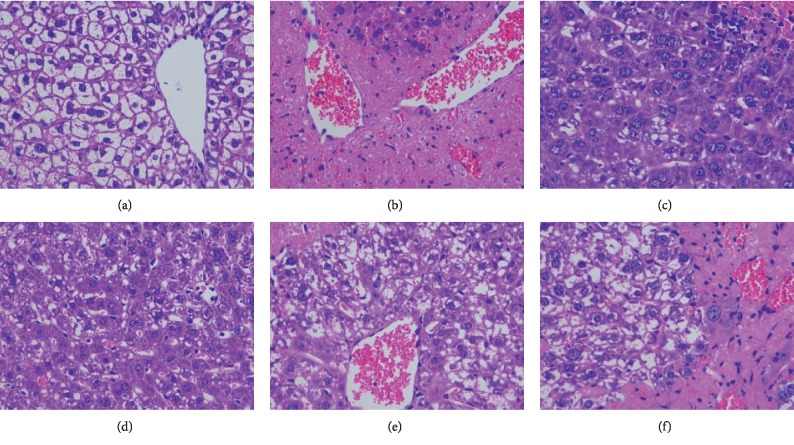
HE staining of liver cells: (a) control, (b) model, (c) silymarin, (d) Dio-NS (high), (e) Dio-NS (medium), and (f) Dio-NS (low).

HE staining showed that the liver cells in the blank control group were arranged normally, the cytoplasm was uniform, the nucleus and nucleolus were obvious, the central vein was clearly visible, and the cells had local edema (Figure 14). In the model group, a large number of necrotic hepatocytes were observed in the tissue, as indicated by a more intense staining or disintegration of liver cells. Furthermore, cytoplasmic eosinophilia was enhanced, accompanied by infiltration of inflammatory cells, and a small amount of white blood cells were seen in the veins. In the silymarin group and the high-dose group of Dio-NS, the structure of hepatocyte was more complete with few inflammatory cells and little necrosis, and the pathological damage was clearly ameliorated. Compared with the model group, the low- and medium-dose groups of Dio-NS decreased the infiltration of inflammatory cells and reduced the degree of hepatocyte damage to a certain extent; however, the effect was not as conspicuous as in the silymarin and high-dose Dio-NS groups.

## 4. Discussion

It is well known that nanoparticle formulations improve the solubility of drugs to some extent because of their small size. However, in the process of drug preparation, excessive heat and solvent residues can adversely affect the production of good formulations. However, dioscin dissolves in ethanol, which has beneficial effects with regards to our preparation, as the rapid volatility of ethanol ensures low amounts of solvent residue.

In previous experiments, we used the reverse solvent preparation method and this method combined with high-pressure homogenization to prepare nanoemulsions. However, when using high-pressure homogenization, we found that the heat generated caused the production of excessive amounts of foam, which we believe may be attributable to the thermal decomposition of surfactants or dioscin. Moreover, using the reverse solvent preparation method alone, we were not able to obtain particles of the requisite size. Therefore, in the present study, we examined the applicability of using magnetic stirring and high-speed shearing combined with high-pressure homogenization to prepare the Dio-NS. Using this approach, the excessive heat generated by homogenization can be dissipated, thereby minimizing or eliminating foaming or degradation of the drug preparation.

Furthermore, we adopted a star design-response surface optimization method in order to determine the optimal formulation ratio. This enabled us to clearly assess each level of interaction. We were surprised to discover that the ratio of dioscin in the formulation was as high as 21.26%.

Through an evaluation of the physical and chemical properties and morphology of Dio-NS, we discovered that the nature of dioscin in the formulation had undergone changes. Observations indicated that the dioscin no longer retained a crystalline form, but instead occurred as spherical particles, as indicated by the disappearance of peaks on thermal analysis.

On the basis of an *in vitro* solubility test, we found that the solubility of dioscin was significantly increased in the Dio-NS formulations. This was confirmed by pharmacodynamic experiments. The possible reasons for these observed properties are as follows. Firstly, the particle size decreases, the adsorption surface is enlarged, the saturation solubility increases, the dissolution rate increases, the permeability of the biofilm increases, at the same time the retention time of the gastrointestinal tract is prolonged, the concentration of drug in the blood is enhanced, and thus the efficacy of the drug is enhanced. Secondly, when drug particles are less than 1 *μ*m in size, the internal dissolution rate will suddenly increase.

However, we did not evaluate the bioavailability of Dio-NS. In this study, we examined the use of ultrahigh liquid phase and liquid mass in combination but were unable to detect any dioscin. On the one hand, dioscin exists in the body in the form of glycogen. On the other hand, there were impertinent methods on handling samples or the type of instruments. These problems will be difficult to solve in subsequent experiments.

## 5. Conclusion

We have demonstrated that nanosuspensions can be successfully prepared using an emulsification method in which high-speed homogenization is used in combination with high-pressure homogenization, which is better than previously prepared drug formulations [32, 33]. Dioscin is an effective component extracted from plants that belong to the family Dioscoreaceae. The present study shows that dioscin has protective effect against acute hepatic injury in mice induced by intraperitoneal injection of CCL_4_. However, dioscin is difficult to dissolve in water, although it can be dissolved in the organic solvent ethanol [34]. Therefore, in this study, we adopted the methods of reverse solvent precipitation and high-pressure homogenization [35] to prepare nanosuspensions. The particle size distribution of the resulting suspensions is between 70 and 120 nm. The suspensions also have a high specific surface area and increased solubility, which may promote their bioavailability.

Therefore, we performed various pharmacodynamic analyses to evaluate the bioavailability of our newly prepared nanosuspensions (Dio-NS). We accordingly found that, at a high dose, Dio-NS reduces the levels of ALT and AST, enhances SOD activity, reduces MDA content, and ameliorates liver pathology to some extent compared with silymarin (the positive control drug).

Thus, the results of the present study will provide a reference for the development and clinical application of dioscin formulations. Finally, in further studies, we intend to evaluate the bioavailability of our dioscin formulations and investigate the precise mechanisms underlying the hepatoprotection properties of these preparations.

## Figures and Tables

**Table 1:** Screening of freeze-drying protectants.

Type	Sensory evaluation	Size (nm)	PDI	Zeta (mv)
Lactose	White powder: shows collapse and shrinkage, uneven color, and poor dispersion.	123.5 ± 0.61	0.278 ± 0.003	−23.2 ± 2.25
Glucose	White powder: shows collapse and shrinkage, uneven color, and poor dispersion	2200 ± 521.85	1 ± 0.001	−42.3 ± 1.86
Sucrose	White powder: shows collapse and shrinkage, uneven color, and poor dispersion.	1195.7 ± 382.27	0.586 ± 0.075	−51.4 ± 5.41
d-sorbitol	White powder: shows collapse and shrinkage, uneven color, and poor dispersion.	172.4 ± 6.99	0.271 ± 0.023	−52.6 ± 2.68
Mannitol	White powder: shows no collapse or shrinkage, uniform color, and good dispersion.	223 ± 1.56	0.273 ± 0.055	−59.1 ± 0.38

(*n* = 3, Mean ± SEM).

**Table 2:** Screening for the dosage of freeze-drying protectants.

Ratio (%)	Size (nm)	PDI	Zeta (mv)
2	433.4	0.620	−40.8
	463.2	0.553	−41.8
	441.3	0.540	−42.8
3	229.2	0.391	−53.4
	231.6	0.402	−58.5
	229.5	0.416	−57.4
5	196.4	0.285	−35.3
	194.8	0.275	−36.9
	194.9	0.285	−36.2
7	173.8	0.234	−53.6
	173.1	0.245	−57.3
	172.7	0.239	−58.0
8	187.4	0.223	−58.0
	182.6	0.245	−57.1
	184.3	0.248	−56.0
10	205.5	0.242	−53.9
	201.6	0.263	−54.2
	207.1	0.272	−56.3

**Table 3:** Stability of Dio-NS freeze-dried powder over a period of 2 months (*n* = 3).

Time (d)	Size (nm)	PDI	Zeta (mV)
0	173.2 ± 0.56	0.235 ± 0.01	−56.3 ± 2.36
15	173.7 ± 0.63	0.237 ± 0.21	−56.54 ± 2.66
30	173.9 ± 0.75	0.237 ± 0.56	−56.73 ± 2.86
60	174.9 ± 0.85	0.239 ± 0.46	−63.43 ± 1.26

**Table 4:** Screening of stabilizers.

Type	Content	MPS	PDI	Macrostability of the system
F-68	0.5%	145.17	0.227	The suspension is white and opaque, producing flocculent precipitates and deepening the color of the system.
SDS	0.5%	125.47	0.196	The suspension is white and opaque and easy to precipitate. It can be dispersed into an original emulsion.
PVP K30	0.5%	91	0.301	The suspension is white and opaque, and it produces microprecipitates, which can be dispersed into an original emulsion.
Tween-80	0.5%	108.8	0.316	The suspension is white and opaque. It is easy to precipitate. It cannot be dispersed into an original emulsion.
Soybean lecithin	0.5%	201	0.269	The suspension is white and opaque, and it produces microprecipitates, which can be dispersed into an original emulsion.
Sodium cholate	0.5%	89.43	0.298	The suspension is white and opaque. It is easy to precipitate. It cannot be dispersed into an original emulsion.
F-68 + SDS	0.25% + 0.25%	175.23	0.294	The suspension is white and opaque, and it produces microprecipitates, which can be dispersed into an original emulsion.
PVP K30 + SDS	0.25% + 0.25%	128.57	0.265	The suspension is white and opaque, and it produces microprecipitates, which can be dispersed into an original emulsion.
Tween + SDS	0.25% + 0.25%	160.5	0.530	The suspension is white and opaque, producing flocculent precipitates and deepening the color of the system.
Lecithin + SDS	0.25% + 0.25%	99.84	0.228	The suspension is white and opaque, and it produces microprecipitates, which can be dispersed into an original emulsion.
F-68 + PVP K30	0.25% + 0.25%	124.2	0.247	The suspension is white and opaque, and it produces microprecipitates, which can be dispersed into an original emulsion.

**Table 5:** Variables and levels in the star design.

Variables	Levels				
	−1.68	−1	0	+1	+1.68
Dio (A)%	0.02	0.03	0.04	0.05	0.06
Sph (B)%	0.03	0.05	0.08	0.10	0.12
SDS (C)%	0.03	0.05	0.08	0.10	0.12

**Table 6:** Response value of the formulation using a central composite design (*n* = 3).

Run	*A*	*B*	*C*	MPS	PDI	OD
1	0	0	0	97.98	0.250	0.661
2	+1	−1	+1	131.45	0.203	0.39
3	0	0	0	97.98	0.250	0.661
4	0	0	0	97.99	0.250	0.661
5	+1	+1	+1	104.9	0.267	0.55
6	−1	−1	−1	85.49	0.226	0.841
7	0	+1.68	0	96.9	0.25	0.67
8	−1	−1	+1	90.29	0.235	0.772
9	−1	+1	+1	106.4	0.27	0.53
10	+1	−1	−1	102.4	0.261	0.59
11	0	0	+1.68	98.23	0.253	0.65
12	0	0	0	97.98	0.250	0.661
13	0	0	0	97.98	0.250	0.661
14	+1.68	0	0	141.6	0.345	0
15	+1	+1	−1	92.93	0.24	0.74
16	−1.68	0	0	108.24	0.274	0.5
17	0	−1.68	0	91.56	0.239	0.75
18	0	0	0	98.97	0.250	0.661
19	0	0	−1.68	74.88	0.238	0.868
20	−1	+1	−1	101.57	0.263	0.59

**Table 7:** Particle size and zeta potential of the optimum Dio-NS (*n* = 3, *x* ± *s*).

Batch no.	Particle size (nm)	PDI	ZP (mV)
VAL1001	106.5 ± 0.50	0.177 ± 0.02	−32.13 ± 2.35
VAL1002	115.73 ± 0.76	0.231 ± 0.002	−33.1 ± 5.26
VAL1003	97.93 ± 1.26	0.254 ± 0.02	−37.6 ± 2.53
Mean value	106.72 ± 8.90	0.221 ± 0.04	−34.27 ± 2.91

## Data Availability

All relevant data are within the paper and its Supporting Information files.

## Abbreviations

Dio: Dioscin
DSC: Differential scanning calorimetry
Dio-NS: Dioscin nanosuspension
E t: Emulsification time
HP: Homogenization pressure
HPH: High-pressure homogenization
HPLC: High-performance liquid chromatography
MPS: Mean particle size
OD: Overall desirability
PSD: Particle size distribution
P s: Passes
SEM: Scanning electron microscope
SDS: Sodium alkyl sulfate twelve
Tem: Transmission electron microscope
XRPD: X-ray powder diffraction
ZP: Zeta potential.

## Supplementary Materials

Supplementary Materials Supplementary Table S1-3: three experimental data during the preparation of nanosuspensions, which describe the effects of shear speed, shear time, temperature, homogenization times, and homogenization pressure on the particle size and polydispersity index of nanosuspensions, and the evaluation results *in vitro* and *in vivo*. The experimental results were plotted by origin 8.5 and GraphPad Prism 5. Supplementary Material 1: Animal Ethics Society approval document.
