# Supplementary material for: Design, Preparation, and Characterization of Dioscin Nanosuspensions and Evaluation of Their Protective Effect against Carbon Tetrachloride-Induced Acute Liver Injury in Mice
Source: Evid Based Complement Alternat Med. 2019 Nov 14;2019:3907915. doi: 10.1155/2019/3907915 (PMC6878791; doi:10.1155/2019/3907915)
Supplement: Supplementary Materials — Supplementary Table S1-3: three experimental data during the preparation of nanosuspensions, which describe the effects of shear speed, shear time, temperature, homogenization times, and homogenization pressure on the particle size and polydispersity index of nanosuspensions, and the evaluation results in vitro and in vivo. The experimental results were plotted by origin 8.5 and GraphPad Prism 5. Supplementary Material 1: Animal Ethics Society approval document. [file 3907915.f1.zip › Supplementary.docx]

The supplementary description and materials as follows:

1."Supplementary table S1-3 "The file contains three experimental data during the preparation of nanosuspensions, which describe the effects of shear speed, shear time, temperature, homogenization times and homogenization pressure on the particle size and polydispersity index of nanosuspensions, and the evaluation results in vitro and in vivo. The experimental results were plotted by origin 8.5 and GraphPad Prism 5.
2."Supplementary Material1. Animal ethics society approval document " The file shows the Animal ethics society approval document.

Please proceed manuscript accordingly
