# Supplementary material for: Design, Preparation, and Characterization of Dioscin Nanosuspensions and Evaluation of Their Protective Effect against Carbon Tetrachloride-Induced Acute Liver Injury in Mice
Source: Evid Based Complement Alternat Med. 2019 Nov 14;2019:3907915. doi: 10.1155/2019/3907915 (PMC6878791; doi:10.1155/2019/3907915)
Supplement: Supplementary Materials — Supplementary Table S1-3: three experimental data during the preparation of nanosuspensions, which describe the effects of shear speed, shear time, temperature, homogenization times, and homogenization pressure on the particle size and polydispersity index of nanosuspensions, and the evaluation results in vitro and in vivo. The experimental results were plotted by origin 8.5 and GraphPad Prism 5. Supplementary Material 1: Animal Ethics Society approval document. [file 3907915.f1.zip › mat.3907915.v2.zip!/Supplementary File/Supplementary Material1.Animal ethics society approval document/Animal ethics society approval document.pdf]

陕西中医药大学实验动物伦理委员会

动物实验伦理审查意见表

<table>
<tr><td rowspan="10">申请人填写的相关信息</td><td colspan="3">申请单位：陕西中医药大学</td></tr>
<tr><td colspan="3">申请人姓名：宋逍　　学历：博士　　技术职称：副教授</td></tr>
<tr><td colspan="3">实验名称：薯蓣皂苷对 CCL4 诱导肝损伤的保护作用</td></tr>
<tr><td colspan="3">实验目的：评价薯蓣皂苷的保肝作用</td></tr>
<tr><td rowspan="4">拟进动物情况</td><td colspan="2">动物来源：成都达硕生物科技有限公司</td></tr>
<tr><td colspan="2">品种品系：昆明小鼠　　等级：SPF 级　　规格：18~22g</td></tr>
<tr><td>数量：48 只（♀ 只；♂48 只）</td><td>申请日期：2018 年 4 月 15 日</td></tr>
<tr><td>进驻日期：　2018 年　4 月 15 日</td><td>结束日期：2018　年 5 月 5 日</td></tr>
<tr><td colspan="3">实验要点，包括实验方法、观测指标、实验结束后处死动物的方法等：采用腹腔注射　CCL4　诱导小鼠肝损伤，采用酶标仪检测血清中　ALT、AST、　TNF-a、IL-1ß、IL-6 和肝组织中 MDA、GSH-px、SOD 的含量。并对肝脏进行 He 染色分析病理结果。实验结束脱颈处死小鼠。</td></tr>
</table>

<table>
<tr><td>项目来源</td><td>国家自然科学基金（No. 81373944，81373978）</td><td>联系电话</td><td>15319015083</td></tr>
</table>

声明：我将自觉遵守实验动物伦理原则，随时接受委员会的监督与检查，如违反规定，自愿接受处罚。

项目负责人签（章）：宋逍

伦理审查意见：☑同意　　☐修改后同意　　☐不同意

审批意见

经初步审核，该项目中所涉及到的动物实验研究将遵照国际公认的动物实验室操作规范和标准操作程序，动物实验方案符合动物保护、动物福利和伦理原则，符合国家实验动物福利伦理的相关规定。

陕西中医药大学实验动物伦理委员会

（盖章）
